# 11509 Data visualization of scholarly productivity data to evaluate the KL2 training programs

**DOI:** 10.1017/cts.2021.575

**Published:** 2021-03-30

**Authors:** Tanha Patel, Beatrice Boateng

**Affiliations:** 1 University of North Carolina at Chapel Hill; 2 University of Arkansas for Medical Sciences

## Abstract

ABSTRACT IMPACT: This work will help assess the effectiveness of the mentored career development programs. OBJECTIVES/GOALS: There is increased attention on assessing the impact of the CTSA in building a research workforce through mentored career development programs. We propose using data visualization to assess and communicate the impact of the programs on the scholars career development. METHODS/STUDY POPULATION: Evaluators from two CTSAs collaborated to visualize the KL2 data such as demographics, scholarly productivity (publications, grants, intellectual property), and time to promotion that is already tracked through REDCap at their institutions. Excel, Tableau, and Microsoft PowerBi were then used to generate trends in scholarly productivity over time. The goal was to compare how different tools can be used to visualize bibliometric data, based on what is available at the respective institutions. RESULTS/ANTICIPATED RESULTS: Longitudinal visual summary reports were produced for the entire program as well individual scholar progress. These reports can be used to identify trends such as how long after program completion do participants achieve their next milestone, what type of milestones are achieved, when in their career is their scholarly productivity the highest, etc. Answers to these questions could tell a story of the effectiveness of a mentored development program in the participants’ career. It can also highlight gaps and areas of opportunities that the program must address, either by adapting their curriculum or clarifying their intended outcomes. DISCUSSION/SIGNIFICANCE OF FINDINGS: Data visualization provides better understanding of the impact of the training programs on the scholars career development. Such insights are otherwise missing when evaluations are only focused on the percentage of scholars who were still engaged in research after completion of the program.

